# The catalytic role of a research university and international partnerships in building research capacity in Peru: A bibliometric analysis

**DOI:** 10.1371/journal.pntd.0007483

**Published:** 2019-07-15

**Authors:** Christopher W. Belter, Patricia J. Garcia, Alicia A. Livinski, Fabiola Leon-Velarde, Kristen H. Weymouth, Roger I. Glass

**Affiliations:** 1 NIH Library, Office of Research Services, National Institutes of Health, Bethesda, Maryland, United States of America; 2 School of Public Health and Administration (FASPA), Universidad Peruana Cayetano Heredia, Lima, Peru; 3 Consejo Nacional de Ciencia, Tecnologia e Innovacion Tecnologica, (CONCYTEC) Lima, Peru; 4 Fogarty International Center, National Institutes of Health, Bethesda, Maryland, United States of America; Johns Hopkins Bloomberg School of Public Health, UNITED STATES

## Abstract

**Objective:**

In Peru, the past three decades have witnessed impressive growth in biomedical research catalyzed from a single research university and its investigators who secured international partnerships and funding. We conducted a bibliometric analysis of publications by Peruvian authors to understand the roots of this growth and the spread of research networks within the country.

**Methods:**

For 1997–2016, publications from Web of Science with at least one author affiliated with a Peruvian institution were examined by year, author affiliations, funding agencies, co-authorship linkages, and research topics.

**Results:**

From 1997–2016, the annual number of publications from Peru increased 9-fold from 75 to 672 totaling 6032. Of these, 56% of the articles had co-authors from the US, 13% from the UK, 12% from Brazil, and 10% from Spain. Universidad Peruana Cayetano Heredia (UPCH) was clearly the lead research institution noted on one-third of publications. Of the 20 most published authors, 15 were Peruvians, 14 trained at some point at UPCH, and 13 received advanced training abroad. Plotting co-authorships documented the growth of institutional collaborations, the robust links between investigators and some lineages of mentorship.

**Conclusions:**

This analysis suggests that international training of Peruvian physician-scientists who built and sustained longstanding international partnerships with funding accelerated quality research on diseases of local importance. The role of a single research university, UPCH, was critical to advance a culture of biomedical research. Increased funding from the Peruvian Government and its Council for Science, Technology and Innovation will be needed to sustain this growth in the future. Middle-income countries might consider the Peruvian experience where long-term research and training partnerships yielded impressive advances to address key health priorities of the country.

## Author summary

One measure of a country’s productivity in biomedical research is through an analysis of the publications in the peer reviewed literature. We have searched the Web of Science database of English language biomedical publications with a Peruvian author to examine the growth in the number of publications over the period 1997–2016, the most productive research institutions in the country, the distribution of foreign coauthors, and the diversity of topics of research. In the past 2 decades, the number of publications has increased 9-fold with the extensive engagement with foreign co-authors and funding, a growing diversity of topics expanding from infectious diseases to the NCDs, and with one third of all publications coming from a single research university. The early overseas training of a group of young Peruvian physician-scientists in the 1960s led them to establish a unique research university, Universidad Peruano Cayetano Heredia that seeded this new direction and engagement in research, and the seeding of researchers to other institutions in the country working on specific research challenges. The Peruvian experience provides a model for other countries seeking to expand their footprint in research while laying out the need for greater investment by the government to secure and expand these gains while retaining outstanding scientists who have been able to identify through research new ways to address the nation’s priority problems in health. The use of bibliometric analyses can provide important insights in the growth of biomedical research in a country providing policy makers with evidence for making decisions on the future funding of research.

## Introduction

In the 1960s, a group of Peruvian physician-scientists who trained abroad returned home with the vision that research should be a fundamental component of medical education. They founded the Universidad Peruana Cayetano Heredia (UPCH) in 1961 and worked with their international collaborators to secure research funding abroad [1]. The success and expansion of these partnerships led not only to the emergence of UPCH as a center of research excellence in Peru but over several decades, to the seeding of their trainees to other institutions around the country where they could address some of the health problems of special concern for Peru, from tropical and viral diseases in the Amazon to studies of the health consequences of populations living at high altitude in the Andes.

Bibliometric methods are frequently used to describe and assess the research capacity of countries and regions [2,3]. In Latin America, for example, regional studies have been performed to assess the region’s research on AIDS [4,5] public health and health policy [6,7,8,9], general medical sciences [10], and all areas of scientific research [11,12]. Specific country-level analyses and comparisons have been performed to assess all scientific research in Brazil [13], health policy in Mexico, Chile, and Argentina (9) and biotechnology in Colombia [14]. We could identify only a single bibliometric study from Peru a decade ago [15].

We have conducted a bibliometric analysis of biomedical publications from Peru over the past two decades to examine the growth of this research in Peru and document key changes in the research enterprise. We wanted to understand the expansion of institutions engaged in research, the emergence of key investigators and their role in mentoring young researchers, the diversification of the topics studied, and the building of international collaborations that has led to success in competing for international grants. This analysis provides insights into public policies that could help stimulate the expansion of biomedical research in the future while assessing its value to the country.

## Methods

### Bibliometric analysis of biomedical research by Peru (1997–2016)

Publications for analysis were obtained from the Web of Science (WoS) Core Collection Database for the 20-year period, 1997–2016 and InCites, an analytical database built on WOS data that automatically calculates a range of bibliometric indicators for every publication in that database. This database is known to have limited coverage of non-English language journals [16,17] but is enough to provide a representative sample of Peruvian publications. Articles were selected when at least one author listed an affiliation with a Peruvian institution that contained a PubMed Identification number (PMID) (Appendix 1: Search Strategy). A series of network analyses were conducted to link coauthors and their institutions with their topics of research. While information on funding organizations was incomplete, we could identify the countries of the foreign coauthors. Although CONCYTEC (Consejo Nacional de Ciencia y Tecnologia- National Council of Science and Technology) was created in 1981 to promote science and technology in Peru, there was no major Peruvian funding agency for research until CONCYTEC in 2014 the program CIENCIACTIVA (active science) that offered grants for biomedical research [18,19].

A bibliographic coupling network was also created to identify key topics. Topic areas in the network were then identified using the Louvain Method [20], an algorithm that identifies groups of articles that share more references with each other than they do with other articles in the network. Topic labels were assigned to each group based on the terms that most frequently appeared in each group and on manual inspection of the publications assigned to each group. All network analyses were performed using the Science of Science (Sci2) Tool [21] and visualized using Gephi [22,23].

## Results

### Bibliometric analyses

**Fig 1:**
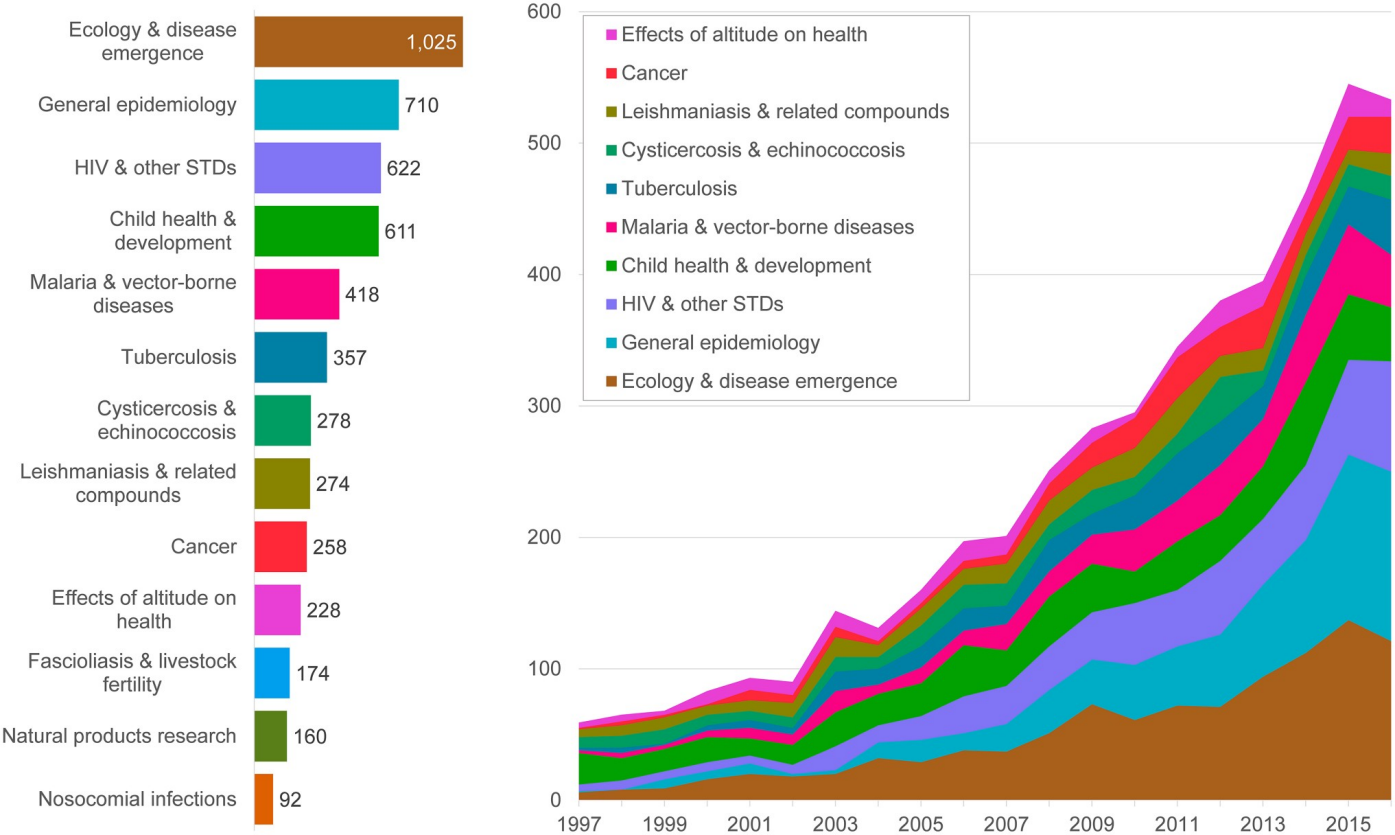
Number of Peruvian publications by year and topic, 1997–2016. Data from WOS, N = 6032.

From 1997 through 2016, 6032 publications were identified from the WOS that met our selection criteria. In this 20-year period, the number increased nearly 9-fold from 75 publications in 1997 to 672 in 2016. (Fig 1). This rapid growth of Peruvian publications is about the same as Iran’s, cited as the highest among the 15 countries with more publications in 2014 and three-fold over the world’s average (6.1% per year). Comparison with global trends actually shows how Peru has grown beyond other countries [24]. Fully two–thirds were on topics of infectious diseases (e.g., parasitology, immunology, tropical diseases, HIV, and microbiology) and the next largest group addressed topics of child, environmental and occupational health and general epidemiology.

**Fig 2:**
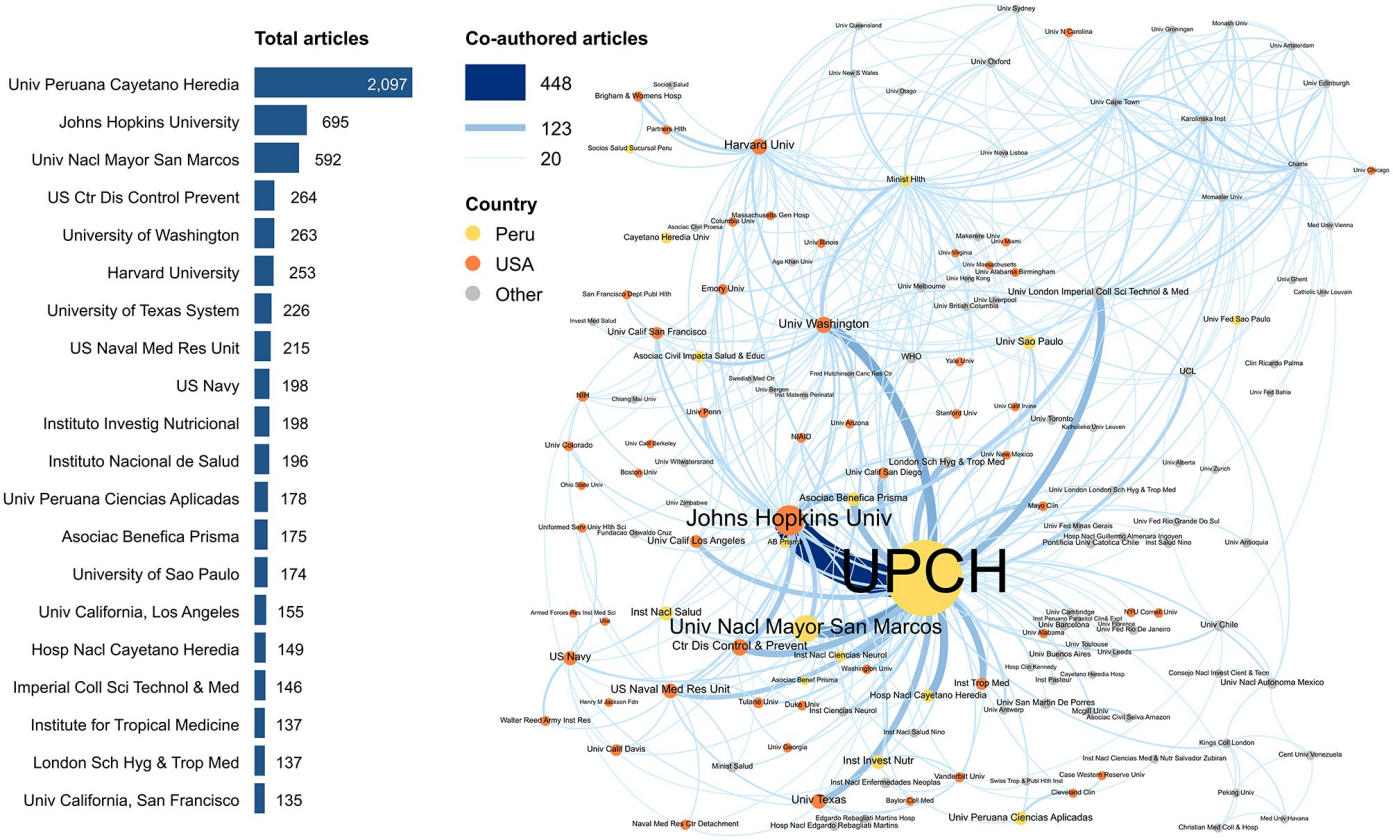
Number of publications per institution and the institutional co-authorship network. Bars indicate the number of publications per institution for the 20 most productive institutions. In the network graphic, circles represent institutions, circle size indicates the institution’s publication count, and circle color indicates whether the institution is located in Peru, the US, or another country. Line thickness and color indicates the number of co-authored publications by authors affiliated with the connected institutions.

An analysis of co-authorship provides insights into how individual investigators were influential in growing the research enterprise over time while building research partnerships and sustaining their international collaborations (Fig 2). In these networking diagrams, the size of the dots and fonts represent graphically the number of publications, the width of the connecting lines represent the extent of these institutional collaborations, and the color of the dots represents the whether the institution is Peruvian, US or other country. UPCH was noted on one third (2097/6032) of all publications with other key hubs at Johns Hopkins University, the Nacional University of San Marcos, Centers for Disease Control and Prevention (CDC) (US), and the University of Washington. Of note, but less common, are the many other collaborations seen between Peruvian institutions and those in neighboring countries and abroad. Overall, 86% of all publications had a foreign co-author, 70% were co-authored with another Peruvian institution, and only 3% included a commercial partner. The most frequent international co-authorships were with investigators from the US (56%), followed by the UK (13%) and Brazil (12%). Of the papers that indicated a source of funding, NIH was noted on 1734 papers (28%) followed by the Wellcome Trust (UK), 312 (5.2%), and the Bill and Melinda Gates Foundation, 139 (2.3%).

The 20 most published authors and their co-author networks provide insights into the importance of mentoring relationships to the growth and expansion of research (Appendix 2). Fifteen of the 20 most published authors were Peruvians and 14 of these received some of their training at UPCH while 13 went on to doctoral or research training abroad. There is a clear spread of researchers beginning their careers at UPCH and moving to other institutions over time with some continuing mentoring relationships with their trainees.

**Fig 3:**
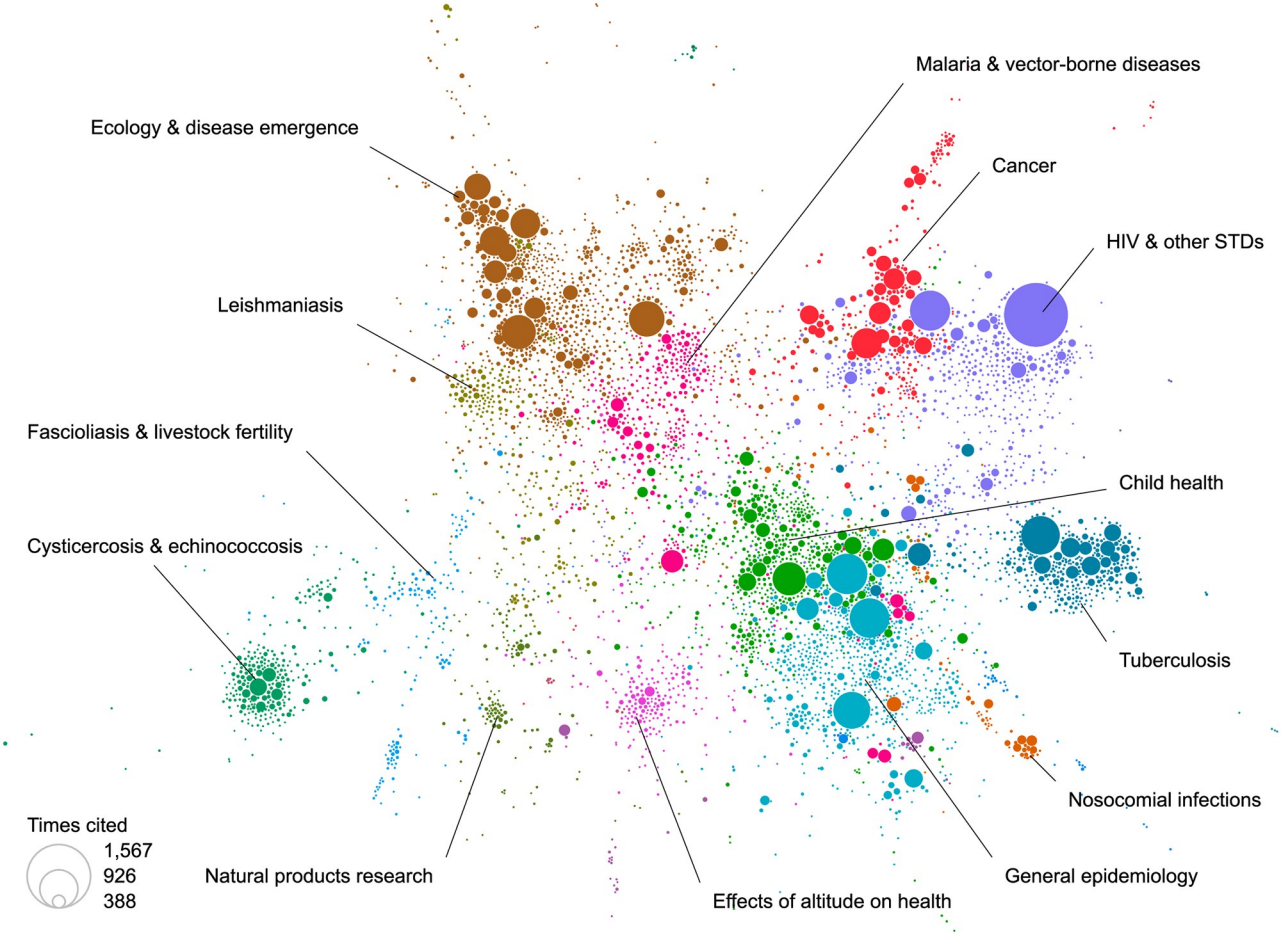
Bibliographic coupling network of Peruvian publications. In this network, circles represent articles, circle size indicates the article’s citation count, circle color indicates the article’s topic, and circle position indicates the article’s similarity to other articles in the network. The largest dot with >1500 citations was a multi-centered international HIV clinical trial where a Peruvian investigator was a co-author.

Major themes of research were examined and plotted using a coupling analysis that linked topics that shared common references (Fig 3). Their proximity on the plot represents how closely the themes were related and the frequency of these citations is represented by the size of the bubble. The most cited papers were large, multi-national clinical trials of HIV and STIs where a Peruvian investigator was a partner. Publications on some topics such as HIV research are linked in the analysis with topics such as cancer, tuberculosis, child health and epidemiology. Others represent themes of local problems- cysticercosis, leishmaniases, malaria, and high-altitude studies- of particular local importance and a draw for international investigators.

Information on grants and awards reported in the database from international funding agencies was incomplete. Since NIH was the largest funder, we were conducted an independent analysis of grants made from NIH to Peru from the iSearch Publications module (Appendix 3). The growth in awards for research in Peru grew over time and matched the growth and diversity of publications with US collaborators. From 2001 to 2016, the NIH awarded 325 unique grants to Peruvian institutions that provided 1130 years of research support. The number of new awards grew from 17 grants in 2001 primarily focused on infectious diseases (from the National Institute of Allergy and Infectious Diseases) and training (the Fogarty International Center), to 74 new grants in 2016 representing 11 NIH institutes and centers on topics ranging from mental health, cancer, neurologic diseases and more.

## Discussion

The growth of biomedical research in Peru over the past two decades is well documented in the nine-fold increase in the number of biomedical articles published in English and the increase in competitive awards from NIH, the largest international funder for which historical information on grants was available. At the same time, the topics of research have expanded to reflect the unique ecology, geography, and disease patterns of the country highlighting parasitic and infectious diseases, high altitude conditions, and infections prevalent in the jungle areas in the Amazon basin. By building the research agenda on diseases and conditions that are priorities in Peru, local investigators have built international collaborations, attracted international funding, and advanced our knowledge of the diagnosis, treatment, and prevention of diseases that are problematic in Peru but less prevalent elsewhere.

A critical finding from this investigation is the central role that a single university can make towards the initiating a culture of research that has trained students for careers in science and for leadership in the biomedical research community. The ability of these groups to collaborate internationally and be competitive for international funding has boosted Peru into the top tier of leadership biomedical research in Latin America. As a result, this research has provided the world with a platform to develop the knowledge to address some of the most compelling problems in global health today that would be hard to study in other settings. Treatments for cysticercosis [25,26], our understanding of high-altitude conditions [27,28] clinical trials of Zika vaccines, policies to address the treatment of resistant tuberculosis [29], and the use of a rapid syphilis test to control congenital disease [30] have all come from research conducted in Peru through these international research collaborations. In addition, the long-term partnerships between Peruvian and foreign investigators has led to mentoring reflected in the network diagrams evident from the bibliometric analyses. Clearly, the value of having outstanding research mentors, both foreign and local, has provided a clear pathway to expand the research workforce in order to address the major health problems in Peru.

This study brought detailed bibliometric analysis to provide insights into the growth of research, the topics of investigation, the mentoring links of investigators, and the centers of excellence followed by the dispersion of groups over time. All of these are helpful for policy makers as one important metric as they decide on priorities for future funding and evaluation of their research enterprise. The use of these the simple metrics of numbers of publications has previously been used to compare countries productivity in research. The high-resolution analysis provides an opportunity to understand the microculture of research in an environment that has evolved rapidly over time.

Our findings raise several questions for policy makers in Peru and elsewhere. For one, given this history of research success in Peru, how can the government become more involved in leveraging their investments for science while training the next generation of researchers for the future? While Peru has many medical schools and universities, UPCH was established as a research university that introduced students to research early in their training, built a culture of research, and encouraged the faculty to seek international funds to support these activities aided by a Vicerector for research at the level of the Vicerector of academic affairs. This structure has been included recently in the new Peruvian law for Universities, highlighting the government’s recognition of the importance of research at academic institutions [31]. The observation that a number of the most prolific investigators have spun off their research units and created clusters of excellence indicates the importance of the dispersion of research to other centers with focus on other topics in other locations apart from UPCH. This has expanded this culture of research to other universities nationwide, such that two thirds of the publications are coming from groups not directly at UPCH.

With improvements in health and longevity, the health problems facing Peru in the future -such as aging of the population, the rise of non-communicable diseases [32], environmental exposures linked to indoor air pollution from cookstoves, use of new diagnostics and information technologies for health, or how to respond to climate change and El Nino events will require even greater diversification of the research portfolio to address these challenges to the populations’ health. The expansion of research funding in Peru and providing a career track for talented young investigators could help leverage even more national and international funding to address some of the world’s most pressing health problems and help Peru achieve the next Sustainable Development Goals. This position has been clearly articulated by the Pan American Health Organization Advisory Committee on Health Research [33]. To date, this bibliometric analysis indicates that work on the chronic diseases has not yet caught up with the key infectious diseases as evidenced by the limited number of publications on these diseases.

This study has limitations, some related to the completeness of the data, the incomplete reporting on international funders, and the fact that our search was focused on publications in English. In addition, while we examined a small group of the 20 authors with the most publications, most of these investigators are themselves training the next generation of researchers, a much larger group for the future that are not fully represented on our network diagrams. Nonetheless, it does provide valuable insights into the growth and dispersion of publications with in depth network diagrams that should permit more nuance understanding of the lineages of research and mentorship that have helped to grow science in Peru.

## Results

The 21^st^ Century will see even greater and more rapid advances in biomedical science and the creation of new knowledge, drugs, vaccines, and interventions to increase our understanding of diseases and ultimately to improve health. Peruvian researchers trained in the recent past have become leaders and policy makers in their health enterprise while they have opened new directions to provide care and extend healthy lives. An economic analysis of the impact of this research on the retention of a skilled workforce in the health sciences, the creation of new jobs and opportunities, and the impact in the lives of people should be a next step. The long-term sustainability of this effort, stimulated in part early on by outside investments, will need to be supplemented through increased local efforts from government, and from the private sector. A national strategy, advancing this research enterprise for the future and investing in infrastructure, human resources and training, could secure the many gains made to date. It could also provide leadership and expansion of research and training throughout the Andean region in populations that share the same health problems and concerns. The bibliometric methods used here are a widely available metric that can serve as one measure of research productivity. The use of this evidence can provide policy makers with insights to develop and assess new policies to stimulate research over time in Peru and beyond.

## Supporting information

S1 Text Search strategy. (DOCX)

S1 Fig Number of publications per author and individual co-authorship network. Bars indicate the number of publications per author for the 20 most productive authors. In the network graphic, circles represent authors, circle size indicates the author’s publication count, and circle color highlights authors with 85 or more publications. Line thickness and color indicates the number of co-authored papers between the connected authors. Five investigators were foreign born (Drs. Gilman, Checkley, and Kochel—US; Dr. Evans–UK; Dr. Arevalo, Bolivia).
(TIF)

S2 Fig Number of active NIH awards with Peruvian institutions, 2000–2016. A total of 325 new grants with 1130 total years of funding were awarded by NIH Institutes and Centers over this period. NIAID–National Institute of Allergy and Infectious Diseases, FIC–Fogarty International Center, NIMH—National Institute of Mental Health, NINDS–National Institute of Neurologic Diseases and Strokes, NICHD–Eunice Kennedy Shriver National Institute of Child Health and Human Development, NCI—National Cancer Institute, and a collection of 5 other institutes. Personal Communication: Dr. Rob Harrison, NIH.
(TIF)
