# Supplementary figures and images for: The catalytic role of a research university and international partnerships in building research capacity in Peru: A bibliometric analysis
Source: PLoS Negl Trop Dis. 2019 Jul 15;13(7):e0007483. doi: 10.1371/journal.pntd.0007483 (PMC6658117; doi:10.1371/journal.pntd.0007483)

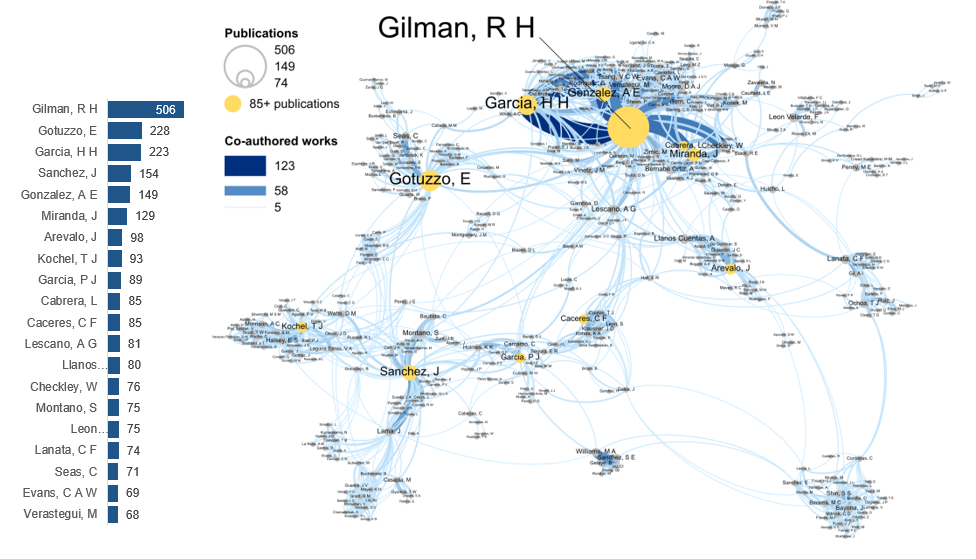

Supplement: S1 Fig — Bars indicate the number of publications per author for the 20 most productive authors. In the network graphic, circles represent authors, circle size indicates the author’s publication count, and circle color highlights authors with 85 or more publications. Line thickness and color indicates the number of co-authored papers between the connected authors. Five investigators were foreign born (Drs. Gilman, Checkley, and Kochel—US; Dr. Evans–UK; Dr. Arevalo, Bolivia). (TIF) [file pntd.0007483.s002.tif]

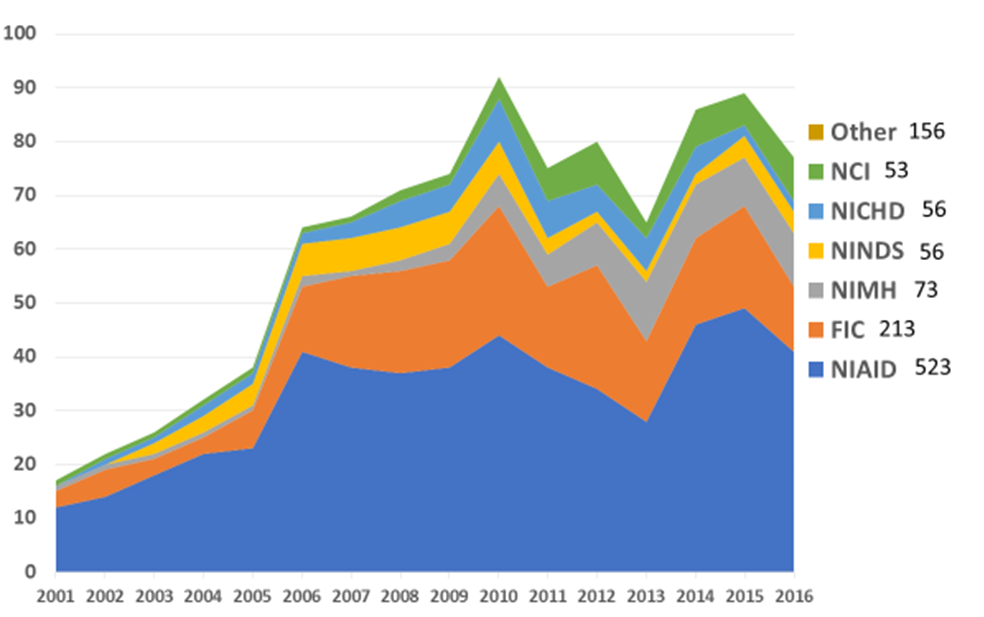

Supplement: S2 Fig — A total of 325 new grants with 1130 total years of funding were awarded by NIH Institutes and Centers over this period. NIAID–National Institute of Allergy and Infectious Diseases, FIC–Fogarty International Center, NIMH—National Institute of Mental Health, NINDS–National Institute of Neurologic Diseases and Strokes, NICHD–Eunice Kennedy Shriver National Institute of Child Health and Human Development, NCI—National Cancer Institute, and a collection of 5 other institutes. Personal Communication: Dr. Rob Harrison, NIH. (TIF) [file pntd.0007483.s003.tif]
